# Semantic categorization of Chinese eligibility criteria in clinical trials using machine learning methods

**DOI:** 10.1186/s12911-021-01487-w

**Published:** 2021-04-15

**Authors:** Hui Zong, Jinxuan Yang, Zeyu Zhang, Zuofeng Li, Xiaoyan Zhang

**Affiliations:** 1 Research Center for Translational Medicine, Shanghai East Hospital, School of Life Sciences and Technology, Tongji University, Shanghai, 200092 China; 2 Philips Research China, Shanghai, 200072 China

**Keywords:** Eligibility criteria, Clinical trials, Semantic category, Clustering, Classification

## Abstract

**Background:**

Semantic categorization analysis of clinical trials eligibility criteria based on natural language processing technology is crucial for the task of optimizing clinical trials design and building automated patient recruitment system. However, most of related researches focused on English eligibility criteria, and to the best of our knowledge, there are no researches studied the Chinese eligibility criteria. Thus in this study, we aimed to explore the semantic categories of Chinese eligibility criteria.

**Methods:**

We downloaded the clinical trials registration files from the website of Chinese Clinical Trial Registry (ChiCTR) and extracted both the Chinese eligibility criteria and corresponding English eligibility criteria. We represented the criteria sentences based on the Unified Medical Language System semantic types and conducted the hierarchical clustering algorithm for the induction of semantic categories. Furthermore, in order to explore the classification performance of Chinese eligibility criteria with our developed semantic categories, we implemented multiple classification algorithms, include four baseline machine learning algorithms (LR, NB, kNN, SVM), three deep learning algorithms (CNN, RNN, FastText) and two pre-trained language models (BERT, ERNIE).

**Results:**

We totally developed 44 types of semantic categories, summarized 8 topic groups, and investigated the average incidence and prevalence in 272 hepatocellular carcinoma related Chinese clinical trials. Compared with the previous proposed categories in English eligibility criteria, 13 novel categories are identified in Chinese eligibility criteria. The classification result shows that most of semantic categories performed quite well, the pre-trained language model ERNIE achieved best performance with macro-average F1 score of 0.7980 and micro-average F1 score of 0.8484.

**Conclusion:**

As a pilot study of Chinese eligibility criteria analysis, we developed the 44 semantic categories by hierarchical clustering algorithms for the first times, and validated the classification capacity with multiple classification algorithms.

## Background

Clinical trials are experiments or observations designed to answer specific clinical scientific questions about biomedical or behavioral interventions and studied on human participants or subject, playing a key role in promoting medical development and improving human health [1, 2]. Eligibility criteria (EC) are fundamental guidelines of clinical trial defined to identify whether a subject meets a clinical trial or not [3], usually written in free text to be human-readable [4]. According to the definition from ClinicalTrials.gov [5], EC are divided into inclusion criteria which are required for a person to participate in the study, and exclusion criteria which prevent a person from participating. The EC text generally describe clinical meaningful characteristics used to determine the eligibility of potential subjects, such as diseases, symptoms, medications, laboratory examinations, demographic characteristics, special population characteristics and informed consent.

The research of eligibility criteria categorization can effectively promote the recruitment of subjects and optimize the design of eligibility criteria. For example, the patients living with human immunodeficiency virus (HIV) or pregnant women are special populations and were excluded by specific defined eligibility criteria in many clinical trials. Zhang et al. [6] developed automatic classification methods for eligibility criteria from clinical trials to facilitate clinical trials recruitment for the criteria describing the specific populations, such as HIV and pregnant women. The 2018 National Natural Language Processing Clinical Challenges (N2C2) [7] focused on automatic diabetic patients recruitment, predefined 13 diabetes specific categories of eligibility criteria, such as “Hba1c” and “Creatinine”, and released 288 complete longitudinal narrative medical records of diabetic patients. It aimed to explore whether it is possible to identify which patient meet eligible criteria by building an automated natural language processing system. The best system achieved highest micro F1 score 0.9100 with a rule-based classifier [8]. These works showed inspiring results for accelerate patients recruitment through parsing different types of eligibility criteria, but overly restrictive eligibility criteria can slow subject recruitment and limit the generalizability of results [9]. The American Society of Clinical Oncology studied the distribution of patients enrolled in clinical trials and real-world patients, and proposed that various types of eligibility criteria should be optimized and the restrictions should be relaxed appropriately [3]. These eligibility criteria contain minimum age [10], HIV-infected patients [11], brain metastases [12], organ dysfunction, prior or concurrent malignancy, and comorbidities [13]. However, most of these work focused on the patient’s special characteristics which are relatively small proportion in the overall eligibility criteria.

The comprehensive research of characteristic categorization of EC text are challenging. Rubin et al. [14] categorized the eligibility criteria of three cancers into 24 categories and developed the tool for authoring new clinical trial protocols based on similarity among eligibility criteria. He et al. [15] collected eligibility criteria from colorectal cancer treatment clinical trials and assessed the population representativeness in quantitative and qualitative aspects respectively. Van Spall et al. [16] selected eligibility criteria from the randomized controlled trials published in high impact journal, and characterized the nature, extend and contribution of 38 categories of exclusion criteria. The BRIDG model [17] defined 17 categories of attributes based on the consensus of domain experts. However these studies proposed eligibility criteria categories only for certain cancers or topics with domain knowledge of biomedical experts, and not applicable to all eligibility criteria of clinical trials. A very challenging problem for characteristic categorization of EC is how to effectively represent the text into vector with the biomedical information. The Unified Medical Language System (UMLS) [18] Metathesaurus shows positive potential to address the challenge. Hao et al. [19] recognized all biomedical concepts of eligibility criteria with MetaMap [20], assigned the UMLS semantic types to these concepts and served as semantic features. As a results, they automatically identified and clustered clinical trials with similar eligibility criteria. Luo et al. [21, 22] downloaded real-world clinical trials eligibility criteria sentences from clinicaltrials.gov, constructed sentence features using UMLS semantic type, and finally obtained 27 types of semantic classes through hierarchical clustering algorithms and manual induction. These works demonstrated the UMLS semantic types can be used for represent the eligibility criteria without losing biomedical information.

To our best knowledge, these works focused on the English eligibility criteria, only an academic conference [23, 24] has paid attention to the Chinese eligibility criteria classification. With the exponential accumulation of Chinese electronic medical records [25] and continued increasing of Chinese clinical trial registration, there is an urgent need to computable characteristic the Chinese eligibility criteria. This research will benefit for knowledge representation [26, 27], cohort definition [28], subject recruitment [29] and clinical decision [30].

In this study, we downloaded clinical trials registration files from the Chinese Clinical Trial Registry (ChiCTR) [31], and extracted Chinese eligibility criteria corpus. We designed the workflow for semantic categories analysis of Chinese eligibility criteria with two steps. First, we implemented hierarchical clustering algorithms for criteria sentence clustering and summarized 44 semantic categories. Second, we validated the classification capacity of our semantic categories with encouraging performance by multiple basic and advanced classification algorithms. As a specific scenario, with proposed categories we can semantically classify the unstructured eligibility criteria, which could facilitate criteria-based clinical trials browsing and retrieval. Furthermore, we can link categorized eligibility criteria to corresponding structured electronic health records to find clinical research opportunities for patients.

## Methods

### Data collection

ChiCTR is a non-profit organization provides the services of register for China’s clinical trials information, it is available in both Chinese and English language. In this study we downloaded clinical trials registration files and extracted the eligibility criteria (EC) text from the sections of “inclusion criteria” and “exclusion criteria” in each trial. There are four types of EC text, including Chinese inclusion criteria (CIC), English inclusion criteria (EIC), Chinese exclusion criteria (CEC), and English exclusion criteria (EEC). An example of eligibility criteria text of Chinese clinical trials showed in Table 1.

**Table 1:** An example of eligibility criteria sentences of Chinese clinical trials registered in ChiCTR

Chinese inclusion criteria	English inclusion criteria
1. 首次于本中心行肝癌切除术, 术后组织病理学证实HCC;	1. Patient receives hepatectomy of HCC, which was confirmed with pathology;
2. 术前血清HBsAg ( +);	2. Serum HBsAg ( +)is confirmed preoperatively;
3. 血清HBV-DNA低于检测下限 (内标法);	3. Serum HBV-DNA is lower than minimum of detection;
4. 肿瘤未侵犯门静脉、肝静脉或胆管的主要分支;	4. Tumor did not invade potal vein,hepatic vein or major branch of biliary tract;
5. 肝功能Child-Pugh A或B级;	5. Liver function with Child–Pugh A or B;
6. 年龄18 ~ 65岁, 性别不限;	6. Aged from 18–65 years male or female;
7. 单发肿瘤者, 最大径 ≤ 5 cm; 多发肿瘤者, 瘤体数 ≤ 3个且各瘤体最大径 ≤ 3 cm;	7. Single tumor ≤ 5 cm.for multiple tumors,number of tumor ≤ 3 and every tumor ≤ 3 cm

Chinese exclusion criteria	English exclusion criteria
1. 肝细胞癌合并肝外转移;	1. HCC with metastasis out of the liver;
2. 近1年内有抗病毒治疗;	2. Antiviral therapy to HBV within one year;
3. 合并其他部位恶性肿瘤, 或其他器官功能衰竭;	3. With other malignancy or organ failure;
4. 合并其它肝炎病毒重叠感染;	4. Combined with other hepatic virus infection;
5. 术前接受TACE或其他抗肿瘤治疗;	5. Recieved TACE or other antitumor therapy;
6. 肝功能Child-Pugh C级	6. Liver function with Child–Pugh C;
7. 存在顽固性腹水或肝性脑病;	7. Refractory Ascites or hepatic encephalopathy;
8. 因精神病、心理性疾病或其他原因不能配合治疗或不能完成疗程者。	8. Patient who can not cooperate due to mental diseases or other reasons

Registration number: ChiCTR1800016069

The EC text is organized as a paragraph with multiple EC sentences. We segmented criteria sentences by the symbol of line break, and excluded the trials in which the number of CIC sentences are not match to the number of EIC sentences, and the number of CEC sentences are not match to the number of EEC sentences. After that, there are 75,754 EC sentence in both Chinese and English remained, and then we filtered the wrong translation and meaningless sentences. Finally we randomly selected 19,185 CEC sentences and EEC sentences in the unsupervised hierarchical clustering section for criteria categories induction, and 38,341 CEC sentences in supervised criteria classification section for the classification capacity validation.

### UMLS semantic types based feature representation

In this section, we described how to convert the Chinese eligibility criteria sentences to UMLS semantic types based feature matrix, perform hierarchical clustering and develop criteria semantic categories.

**Fig. 1:**
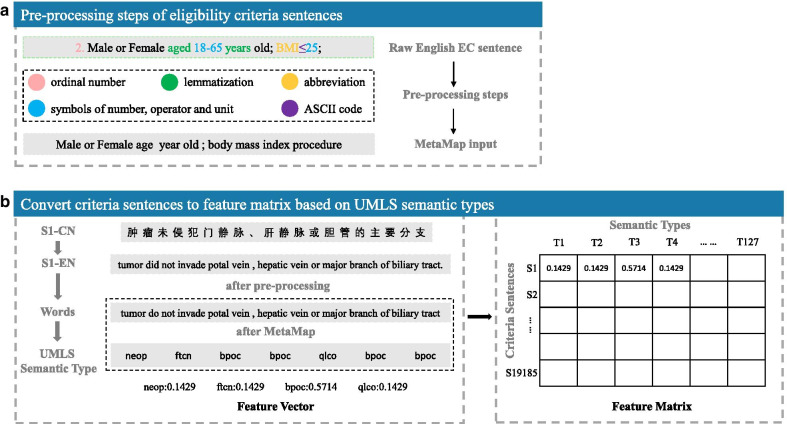
Pipeline of eligibility criteria processing and clustering. **a** An English EC sentence pre-processing demonstration example. **b** The process of transform Chinese eligibility criteria into feature matrix based on UMLS semantic types. neop, Neoplastic Process; ftcn, Functional Concept; bpoc, Body Part, Organ, or Organ Component; qlco, Qualitative Concept

Because of the EC sentences are written by the designer or leader of clinical trials, there are a variety of different criteria expressions, even some wrong writings and ASCII code especially for English EC. We performed a series of pre-processing steps to normalize the criteria sentences, including delete ordinal number, delete the ASCII code, lemmatization, replace the abbreviation, and delete symbols of number, operator and unit. Figure 1a was a criteria sentence example for pre-processing steps demonstration, and Table 2 showed the detailed descriptions of these pre-processing steps.

**Table 2:** The descriptions of pre-processing steps of English eligibility criteria sentences

English eligibility criteria sentences preprocess	Descriptions
Delete ordinal number	There are many types of ordinal number (e.g., “1.”, “①”, “(1)”), and were deleted by regular expression
Replace the ASCII code	We replace the ASCII code with the format that MetaMap can handle based on rules
Lemmatization	Lemmatization is a process of grouping together the different inflected forms of a word and be analyzed as canonical form of the word. We did it with Python package NLTK
Replace abbreviation	We replace the abbreviation with full spelling format based on dictionary
Delete symbols of number, operator and unit	Various expression formats of number, operator and unit sometimes will interfere the output of MetaMap, and was deleted by regular expression

Figure 1b showed the process of transform a Chinese EC sentence into UMLS semantic types based feature vector. For a Chinese EC sentence *S1-CN*, we first obtained the corresponding English EC sentence *S1-EN*. Then MetaMap [20], a highly configurable natural language processing tool, was applied to process *S1-EN* for identifying the UMLS semantic types. The values of the feature vector were calculated by frequency. Finally, all EC sentences were processed and transformed into a semantic feature matrix, where the row represents 127 UMLS semantic types and the column represents 20,000 criteria sentences.

### Hierarchical clustering and semantic categories induction

We applied the agglomerative hierarchical clustering algorithm with “bottom-up” manner to cluster constructed semantic feature matrix and generated clusters based on criteria sentences similarity. Hierarchical clustering is a tree based clustering and it is easy to choose the parameters. We used hierarchical clustering to perform human–computer interaction for identifying categories and labeling ground truth. It starts by treating each criteria sentence as a separate cluster, and then merges two clusters that most closest based on distance similarity measurement into one cluster. Repeat until only a single cluster remains. In order to better summarize categories, we involved two biomedical researchers reviewed the clustering results, merged similar clusters by judging similarity of their criteria sentences expressions, and generalized the semantic categories.

We implemented the algorithm using Python library scikit-learn version 0.24.0. The parameters for sentences similarity measure was set to Euclidean, clusters similarity measure was set to Average Linkage Method. Distance threshold means the minimum similarity of criteria sentences in one cluster. The high distance threshold would generate a few large clusters, while a low distance threshold would generate many small clusters. We set the threshold to 0.65.

### Eligibility criteria classification with multiple algorithms

To assess the classification capacity of our developed semantic categories used for the Chinese eligibility criteria sentences classification, we randomly selected 38,341 Chinese eligibility criteria sentences. One researcher (Z.L) and two raters (Z.Z and J.Y) of biomedical domains labeled the corpus with the semantic categories. First, they studied these categories definition, investigated a large amount of expression patterns of criteria sentences, and chose criteria examples of each category. Next, the two raters independently annotated same 1000 sentences, then they checked annotations and discussed contradictions with Z.L until consensus was achieved. This step repeated 20 iterations and 20,000 criteria sentences were annotated which were later used to calculate the inter-annotator agreement score. Finally, remaining 18,341 sentences were assigned to the two raters for annotation.

We implemented multiple classification algorithms in this section, including machine learning algorithms, deep learning algorithms and pre-trained language models. Four machine learning classification algorithms: k nearest neighbor (kNN), logistic regression (LR), support vector machine (SVM), and naïve Bayesian (NB) were set as our baseline classifiers, and developed by Python version 3.6.9 (scikit-learn version 0.24.0 [32]). We converted criteria sentence to 768 dimensions feature vector representation using bert-as-service version 1.9.6 [33]. A grid search algorithm GridSearchCV with threefold cross-validation was performed on training data to optimize the parameters. For kNN model, we optimized the parameter n_neighbors = 8 (which was tested from 2 to 15). For LR model, we set parameter solver as ‘liblinear’ and optimized parameter C = 1e0 (which was tested from 1e−4 to 1e1). For SVM model, we set parameter kernel as default and optimized parameters C = 1e1 (which was tested from 1e−2 to 1e2) and gamma = 1e−2 (which was tested from 1e−3 to 1e1). For NB model, we used Gaussian Naive Bayes model with default parameters. The selected best parameters were then applied to evaluate the classification performance in our test data.

The deep learning algorithms include Convolutional Neural Network (CNN) for sentence classification proposed by Kim [34], Recurrent Neural Network (RNN) and FastText [35]. In the data preprocessing, we built a dictionary based on all criteria sentences to map each character to a corresponding numerical value, and used the first 50 characters in each sentence for training. In CNN model, we applied cross entropy loss function and Adam optimizer, batch size was 128 and learning rate was 1e-3. We performed dropout on the concatenated filter outputs and passed them through a linear layer to make predictions. In RNN model, we implemented a bidirectional stacked long short-term memory (LSTM) model, and a fully connected layer with softmax activation was used to predict classification results. We applied Adagrad optimizer, batch size was 256 and learning rate was 2e−3. In FastText, the embedding is associated with character n-grams and used for word representation and sentence classification. We implemented FastText model by Python library fasttext (version 0.9.2), and used its automatic hyperparameter optimization function.

The pre-trained language models BERT [36] and ERNIE [37] were fine-tuned on the training dataset and evaluated on testing dataset respectively. In preprocessing, we first added “[CLS]” token at the beginning, and “[SEP]” token at the end of each input text respectively. Second, we added the padding token “[PAD]” to sentences to make up the max length. In our implementation, we set max length as 50 due to most criteria sentences are short and 90% sentences in our corpus with length less than or equal to 50 characters. Finally, we converted criteria sentence into numerical vector by mapping each character to its corresponding unique value. In training, we applied cross entropy loss as loss function and AdamW optimizer, the learning rate was 2e-3, batch size was 128, and ran 10 rounds. Finally we used a fully connected layer to output the classification probability results. The two pre-trained language models were developed based on Python version 3.6.9 (PyTorch version 1.7.1) and open source pre-trained parameters.

For the reproducibility of our results, we fixed the random number seed to 2021. A Tesla P100 graphics card with 12 GB memory size was used.

To measure classification performance of each semantic category, we calculated the basic classification metrics: precision, recall and F1 score. The formulas were given below, for our *n* semantic categories: $C_{1}$, …, $C_{i}$, …, $C_{n}$,

the precision of category *i* is defined as:

1 $$Precision^{i} = \frac{{The \;number\;of\;samples\;correctly\;predicted\;as\;C_{i} }}{{the\;number\;of\;samples\;predicted\;as\;C_{i} }}$$

the recall of category *i* is defined as:

2 $$Recall^{i} = \frac{{The\;number\;of\;samples\;correctly\;predicted\;as\;C_{i} }}{{the\;number\;of\;samples\;of\;C_{i} }}$$

the F1 score of category *i* is defined as:

3 $$F1{ - }score^{i} = \frac{{2*Precision^{i} *Recall^{i} }}{{Precision^{i} + Recall^{i} }}$$

Furthermore, these metrics were averaged across *n* semantic categories in both macro and micro levels to compare the overall performance of these classification algorithms.

## Results

### Eligibility criteria semantic categories

We set the distance threshold as 0.65, and 295 clusters generated. Then we merged similar clusters and summarized 44 semantic categories. Table 3 shows the 44 semantic categories of Chinese clinical trials eligibility criteria and their distributions in the 19,185 criteria sentences corpus used for hierarchical clustering. *Disease (23.40%)* and *Multiple (19.29%)* are the most frequent semantic categories, covering 42.69% of the Chinse eligibility criteria sentences. The other high frequent categories include *Therapy or Surgery (6.96%)*, *Consent (5.74%)*, *Diagnostic (5.19%)*, *Laboratory Examinations (4.93%)*, and *Pregnancy-related Activity (4.65%)*. The *Multiple* is a special category in Chinses eligibility criteria. It is generally long sentence with complex expression and information. In addition, we generalized the 44 semantic categories into 8 topic groups include *Health Status (36.11%), Treatment or Health Care (11.20%), Diagnostic or Lab Test(13.02%), Demographic Characteristics (5.02%), Ethical Consideration (12.73%), Lifestyle Choice (2.01%), Data or Patient Source (0.64%),* and *Others (19.29%).*

**Table 3:** The summarized 8 topic groups and 44 criteria categories of Chinese eligibility criteria, as well as ratio (count) in 19,185 criteria sentences, average incidence and prevalence in 272 HCC related clinical trials

Topic group	Criteria category	Ratio (count)	Average incidence	Prevalence
Health status	Disease	23.40% (4489)	2.7904 (759)	68.75% (187)
	Symptom	0.36% (70)	0.0110 (3)	1.10% (3)
	Sign	1.64% (314)	0.0294 (8)	2.94% (8)
	Pregnancy-related activity	4.65% (893)	0.4118 (112)	33.82% (92)
	Neoplasm status	0.14% (26)	0.1397 (38)	11.03% (30)
	Non-neoplasm disease stage	0.62% (118)	0.0074 (2)	0.74% (2)
	Allergy intolerance	2.80% (538)	0.2132 (58)	19.49% (53)
	Organ or tissue status	1.67% (321)	0.2574 (70)	21.32% (58)
	Life expectancy	0.66% (127)	0.1838 (50)	17.65% (48)
	Oral related	0.16% (31)	0.0000 (0)	0.00% (0)
Treatment or health care	Pharmaceutical substance or drug	3.77% (724)	0.1213 (33)	9.93% (27)
	Therapy or surgery	6.96% (1336)	1.2463 (339)	54.78% (149)
	Device	0.40% (77)	0.0221 (6)	2.21% (6)
	Nursing	0.06% (12)	0.0000 (0)	0.00% (0)
Diagnostic or lab test	Diagnostic	5.19% (995)	0.6103 (166)	40.81% (111)
	Laboratory examinations	4.93% (945)	0.8934 (243)	29.41% (80)
	Risk assessment	2.86% (549)	0.7757 (211)	42.65% (116)
	Receptor status	0.04% (8)	0.0074 (2)	0.74% (2)
Demographic characteristics	Age	4.12% (790)	0.2353 (64)	22.79% (62)
	Special patient characteristic	0.31% (59)	0.0074 (2)	0.74% (2)
	Literacy	0.17% (32)	0.0000 (0)	0.00% (0)
	Gender	0.11% (21)	0.0000 (0)	0.00% (0)
	Education	0.07% (14)	0.0000 (0)	0.00% (0)
	Address	0.17% (32)	0.0110 (3)	1.10% (3)
	Ethnicity	0.08% (15)	0.0000 (0)	0.00% (0)
Ethical consideration	Consent	5.74% (1101)	0.4632 (126)	38.60% (105)
	Enrollment in other studies	2.35% (451)	0.1176 (32)	11.76% (32)
	Researcher decision	1.98% (379)	0.1324 (36)	12.50% (34)
	Capacity	0.73% (140)	0.0184 (5)	1.84% (5)
	Ethical audit	0.02% (3)	0.0037 (1)	0.37% (1)
	Compliance with protocol	1.92% (368)	0.2022 (55)	17.65% (48)
Lifestyle choice	Addictive behavior	1.27% (244)	0.0221 (6)	2.21% (6)
	Bedtime	0.02% (4)	0.0000 (0)	0.00% (0)
	Exercise	0.10% (20)	0.0000 (0)	0.00% (0)
	Diet	0.24% (46)	0.0000 (0)	0.00% (0)
	Alcohol consumer	0.05% (10)	0.0000 (0)	0.00% (0)
	Sexual related	0.02% (3)	0.0000 (0)	0.00% (0)
	Smoking status	0.22% (42)	0.0000 (0)	0.00% (0)
	Blood donation	0.08% (16)	0.0000 (0)	0.00% (0)
Data or Patient source	Encounter	0.22% (43)	0.0000 (0)	0.00% (0)
	Disabilities	0.02% (4)	0.0000 (0)	0.00% (0)
	Healthy	0.11% (22)	0.0000 (0)	0.00% (0)
	Data accessible	0.28% (53)	0.0294 (8)	2.94% (8)
Others	Multiple	19.29% (3700)	1.4632 (398)	78.68% (214)

To comprehensively understand the 44 semantic categories in Chinese eligibility criteria, we investigated two metrics defined by Luo [22], the average incidence and trial prevalence. The average incidence was defined as the average criteria sentence number of each semantic category in a clinical trial study, and trial prevalence was the percentage of trials that containing criteria sentence of a particular semantic category. We selected 272 Hepatocellular carcinoma (HCC) related clinical trials to calculated the two metrics. As shown in Table 3, *Disease* is the most popular category, it appeared 2.79 times on average in each HCC trial and mentioned by 68.75% HCC trials. Other semantic categories such as *Therapy and Surgery*, *Risk Assessment*, *Diagnostic*, *Consent*, and *Pregnancy-related Activity* have high prevalence ranged from 33.82% to 54.78%. The semantic categories in topic of *Demographic Characteristics* (exclude *Age*) and *Lifestyle Choice* (exclude *Addictive Behavior*) are no occurrence in HCC trials.

### Alignment of our semantic categories with previous studies

**Fig. 2:**
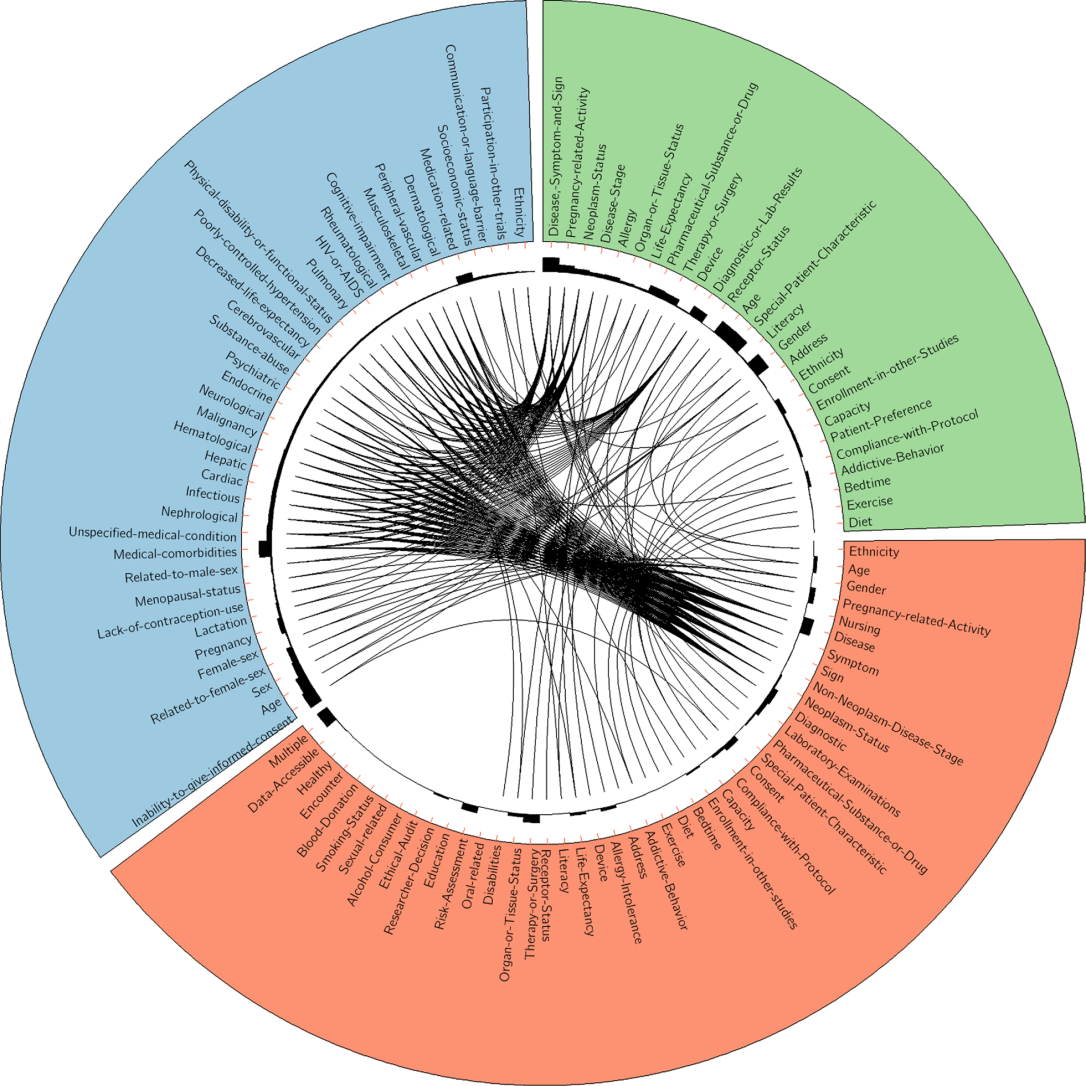
Comparison of our proposed semantic categories with the previous works. The alignment of our 44 semantic categories (green) with Luo’s 27 semantic classes (red) and Van Spall’s 38 categories (blue). The histograms represent the prevalence. In Luo’s work, prevalence was calculated with 1578 randomly selected clinical trials from www.clinicaltrials.gov. In Van Spall’s work, prevalence was calculated with 283 random clinical trials published in high impact medical journals. In our study, prevalence was calculated with 272 HCC related clinical trials from ChiCTR. The lines represent the two categories have same meaning. The 13 semantic categories not linked with any other categories, are novel categories in Chinese eligibility criteria

We compared our semantic categories of Chinese eligibility criteria with three previous studies of English eligibility criteria, including Luo’s 27 semantic classes [22] and Van Spall’s 38 categories [16]. As shown in Fig. 2, luo’s 27 semantic classes are induced from a large number of randomly selected English eligibility criteria, and all covered by our semantic categories. The Van Spall’s 38 categories are summarized from 283 random clinical trials published in high impact medical journals. We have aligned 37 out of 38 categories with our semantic categories, except the “*Socioeconomic status*”. In addition, we also defined 13 novel semantic categories with the total distribution of 25.36% in Chinese eligibility criteria.

### Classification capacity validation

**Fig. 3:**
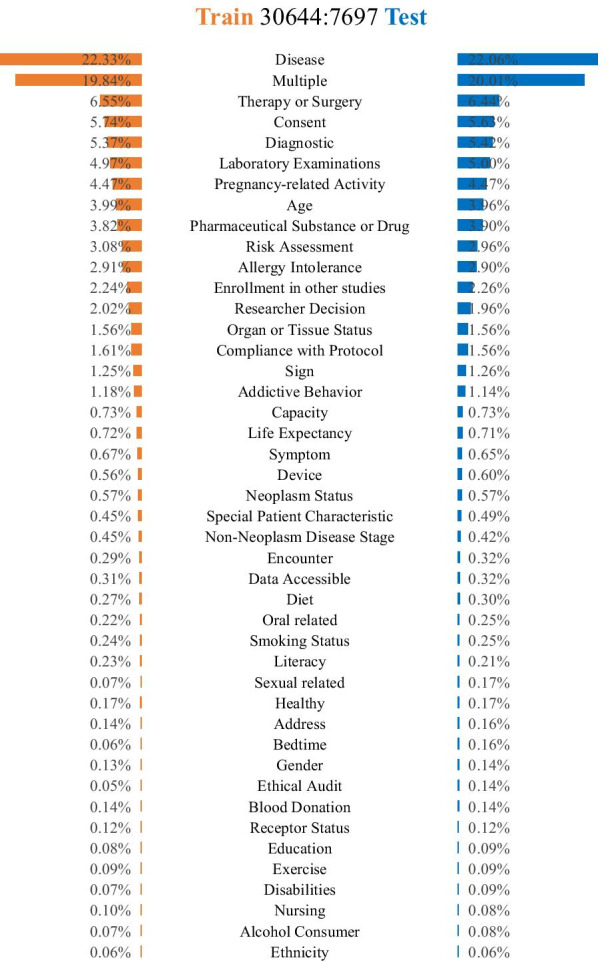
The detailed data distribution of each semantic category for training and testing

A total of 38,341 Chinese eligibility criteria sentences were randomly selected and annotated. The two annotators achieved 0.9920 inter-annotator agreement score by Cohen’s kappa. Among these Chinese criteria sentences, 30,644 (80%) were used to train the classifiers and 7697 (20%) were used for testing. The detailed data distribution of each semantic category for training and testing are shown in Fig. 3.

**Fig. 4:**
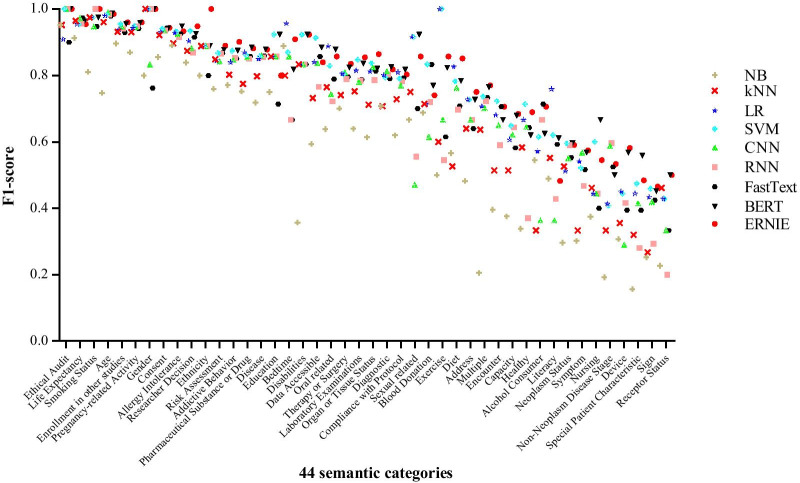
The F1 score of each semantic category by 9 classifiers

We implemented three types of classification algorithms, including machine learning algorithms (NB, kNN, LR, SVM), deep learning algorithms (CNN, RNN, FastText), and pre-trained language models (BERT, ERNIE). As shown in Fig. 4, the abscissa represents 44 types of semantic categories, the ordinate represents the F1 score of each semantic categories of 9 classifiers, we ranked the semantic categories by the average F1 scores. Most of semantic categories achieved F1 score values above 80%, the category with best performance is *Ethical Audit* with F1 score nearly 100% by all 9 classifiers. Other categories such as *Life Expectancy, Smoking Status, Age, Enrollment in other studies, Pregnancy-related Activity, Gender, Consent, and Allergy Intolerance* achieved the average F1 score of 9 classifiers above 90%. Some categories achieved 100% F1 score by some classifiers, such as *Smoking Status* by ERNIE and RNN, *Ethical Audit* by SVM, CNN, RNN, BERT and ERNIE, *Gender* by kNN, LR, SVM, RNN, BERT and ERNIE, *Ethnicity* by ERNIE, *Exercise* by LR and SVM.

Furthermore, we compared the overall performance among these classifiers by average precision, recall and F1 score in both macro and micro levels. The results are shown in Table 4, the pre-trained language models perform significantly better than machine learning algorithms and deep learning algorithms, the ERNIE achieved best results with macro-average F1 score of 0.7980 and micro-average F1 score of 0.8484.

**Table 4:** the overall classification performance comparison of 9 classifiers averaged in macro and micro level

Models	Macro-average			Micro-average		
	Precision	Recall	F1-score	Precision	Recall	F1-score
Machine learning algorithms						
NB	0.5398	0.7403	0.5965	0.6312	0.6312	0.6312
kNN	0.7531	0.6693	0.6948	0.7632	0.7632	0.7632
LR	0.8017	0.7574	0.7732	0.8173	0.8173	0.8173
SVM	**0.8196**	0.7712	0.7899	0.8293	0.8293	0.8293
Deep learning algorithms						
CNN	0.8004	0.6951	0.7258	0.8142	0.8142	0.8142
RNN	0.7837	0.6925	0.7170	0.8138	0.8138	0.8138
FastText	0.7645	0.7188	0.7341	0.8182	0.8182	0.8182
Pre-trained language models						
BERT	0.7994	0.8023	0.7958	0.8447	0.8447	0.8447
ERNIE	0.7964	**0.8074**	**0.7980**	**0.8484**	**0.8484**	**0.8484**

Bold indicates the best value per metric

## Discussion

In this study, we comprehensively characterized semantic categories of Chines eligibility criteria of clinical trials for the first time. Two aspects demonstrated the notable contribution of our work. First, we developed 44 semantic categories of Chinese eligibility criteria by hierarchical clustering algorithms with 127 UMLS semantic types based feature representation, and generalized 8 topic groups. Second, we validated the classification capacity of the semantic categories with encouraging performance by multiple basic and advanced classification algorithms.

Text clustering is an unsupervised learning approach to partitioning unlabeled text data into meaningful groups with similar data [38], generally used for mining valuable information, such as the categories. Such task often relies on text feature representation and vector dimension reduction. In this study, we represented criteria sentence into feature vector with 127 dimensions based on UMLS semantic types, which provide a consistent categorization of all biomedical concepts in the UMLS metathesaurus. We utilized UMLS semantic types here for two reasons. First, the criteria sentences with same meaning often have similar writing in English but diverse writing in Chinese. For instance, the sentences “性别不限 (male or female)” and “男女均可 (male or female)” contain consistent information with almost same words in English but totally different words in Chinese. Second, there are a large number of concepts in criteria sentences with various names but similar semantic information. For instance, the criteria “肌酐清除率(creatinine clearance)” and “白细胞计数 (white blood cell count)” are both represent laboratory examination items. So it is hard to cluster these criteria sentences into one group based on common feature representation methods such as one-hot encoding, bag-of-words, or N-gram models. Fortunately, with the help of corresponding English criteria, we can get the UMLS semantic types, *Qualitative Concept* for “male”, *Population Group* for “female”, *Laboratory Procedure* for “creatinine clearance” and “white blood cell count” and thus easily cluster the criteria sentences. Moreover, the UMLS-based feature representation method contains rich biomedical semantic information with low dimension.

We also compared our semantic categories of Chinese eligibility criteria with multiple existing categories developed in English eligibility criteria. The result shown that most previous categories can be covered by our semantic categories, which suggest that most common data elements are shared between Chinese and English eligibility criteria. We also defined 13 novel semantic categories which not specified before but high prevalence. For instance, the *Risk Assessment* has 42.49% prevalence in Chinese HCC related clinical trials. Many long and complex criteria have multiple short atomic sentences and thus cannot be assigned to a single category, we named this type of criteria as *Multiple*. It should be further optimized for physicians to clearly explain to patients [39] and easily determine patient’s eligibility [40]. These results imply that Chinese eligibility criteria are generally more diverse and complex.

Automatic classification of eligibility criteria are foundational task for advanced downstream tasks of eligibility criteria analysis and applications, such as criteria information structure [27], subject eligibility identify [41], and automatic patient screening [30]. In this study, we demonstrated the classification of Chinese eligibility criteria with multiple algorithms, and achieved encouraging performance. However, there are some limitations need to be addressed in the future. First, the performance is restricted by the special characteristic words and imbalance data distribution of semantic categories, and this is related with the average incidence and prevalence in clinical trials. For instance, *Age*, *Smoking Status* and *Life Expectancy* have relatively small data but achieved best F1 score, because words in these criteria are generally specific and discriminative. *Pregnancy-related Activity*, *Allergy Intolerance* and *Disease* contain various medical concept in the criteria but achieved relatively high F1 scores because of larger data volume. Second, we did not preprocess the Chinese eligibility criteria before training the classifiers. In addition, we observed that there are many special characters in sentences, such as special expression (symbols of number, operator, unit), stop words, traditional Chinese characters and full-width characters. Thus our future work will focus on improving classification performance by preprocess text data of some special characters.

Furthermore, a shared task in the fifth China Conference on Health Information Processing (CHIP 2019) [24] were organized to expand the 44 semantic categories and our developed methods to more domain researchers. As organizers, we released our labeled data and set our classification results as reference. A total of 75 teams participated in the task and 27 of them submitted results. The best performing system achieved a macro F1 score of 0.81 by using multiple pre-trained language models and ensemble modeling. We believe that this study could provide a valuable dataset and comparable results for the domain of clinical trials eligibility criteria research, as well as Chinese medical short text classification.

## Conclusions

In summary, this study explored the semantic categories analysis of Chinese eligibility criteria in two aspects. First, we implemented unsupervised hierarchical clustering algorithms on Chinese eligibility criteria and developed 44 semantic categories for the first time. Compared with previous researches of English eligibility criteria, we defined 13 novel semantic categories. Second, we implemented multiple supervised classification algorithms on automatic Chinese eligibility criteria classification. Most semantic categories showed encouraging performance, and the pre-trained language model ERNIE achieved best results with macro-average F1 score of 0.7980 and micro-average F1 score of 0.8484. We believe that our study provide valuable information for understanding Chinese eligibility criteria of clinical trials.

## Data Availability

The datasets and scripts used during the current study are available in the GitHub repository at the URL: https://github.com/zonghui0228/SemCat-CECCT.

## Abbreviations

EC: Eligibility criteria
HIV: Human immunodeficiency virus
UMLS: Unified Medical Language System
ChiCTR: Chinese Clinical Trial Registry
CIC: Chinese inclusion criteria
EIC: English inclusion criteria
CEC: Chinese exclusion criteria
EEC: English exclusion criteria
NB: Naïve Bayesian
kNN: K nearest neighbor
LR: Logistic regression
SVM: Support vector machine
CNN: Convolutional neural network
RNN: Recurrent neural network
HCC: Hepatocellular carcinoma
